# Synergistic Effect of Omega-3 Fatty Acids and Oral-Hypoglycemic Drug on Lipid Normalization through Modulation of Hepatic Gene Expression in High Fat Diet with Low Streptozotocin-Induced Diabetic Rats

**DOI:** 10.3390/nu12123652

**Published:** 2020-11-27

**Authors:** Suresh Khadke, Pallavi Mandave, Aniket Kuvalekar, Vijaya Pandit, Manjiri Karandikar, Nitin Mantri

**Affiliations:** 1 Interactive Research School for Health Affairs, Bharati Vidyapeeth, Deemed to be University, Pune-Satara Road, Pune 411043, Maharashtra, India; spkhadke@gmail.com (S.K.); mandavepallavi@gmail.com (P.M.); kuaniket@gmail.com (A.K.); 2 Department of Pharmacology, Bharati Vidyapeeth Medical College, Bharati Vidyapeeth, Deemed to be University, Pune-Satara Road, Pune 411043, Maharashtra, India; vijaya.pandit@bharatividyapeeth.edu; 3 Department of Pathology, Bharati Vidyapeeth Medical College, Bharati Vidyapeeth, Deemed to Be University, Pune-Satara Road, Pune 411043, Maharashtra, India; manjiri.karandikar@bharatividyapeeth.edu; 4 The Pangenomics Lab, School of Science, RMIT University, Melbourne, VIC 3000, Australia

**Keywords:** type 2 diabetes mellitus, glibenclamide, omega-3 fatty acids, high fat diet, transcription factors, streptozotocin

## Abstract

Type 2 diabetes mellitus, which an outcome of impaired insulin action and its secretion, is concomitantly associated with lipid abnormalities. The study was designed to evaluate the combinational effect of omega-3 fatty acids (flax and fish oil) and glibenclamide on abnormal lipid profiles, increased blood glucose, and impaired liver and kidney functions in a high fat diet with low streptozotocin (STZ)-induced diabetic rats, including its probable mechanism of action. The male Wistar rats (*n* = 48) were distributed into eight groups. All animal groups except the healthy received a high fat diet (HFD) for 90 days. Further, diabetes was developed by low dose STZ (35 mg/kg). Diabetic animals received, omega-3 fatty acids (500 mg/kg), along with glibenclamide (0.25 mg/kg). Both flax and fish oil intervention decreased (*p* ≤ 0.001) serum triglycerides and very low density lipoprotein and elevated (*p* ≤ 0.001) high density lipoprotein levels in diabetic rats. Total cholesterol and low-density lipoprotein level was decreased (*p* ≤ 0.001) in fish oil-treated rats. However, it remained unaffected in the flax oil treatment group. Both flax and fish oil intervention downregulate the expression of fatty acid metabolism genes, transcription factors (sterol regulatory element-binding proteins-1c and nuclear factor-κβ), and their regulatory genes i.e., acetyl-coA carboxylase alpha, fatty acid synthase, and tumor necrosis factors-α. The peroxisome proliferator-activated receptor gamma gene expression was upregulated (*p* ≤ 0.001) in the fish oil treatment group. Whereas, carnitine palmitoyltransferase 1 and fatty acid binding protein gene expression were upregulated (*p* ≤ 0.001) in both flax and fish oil intervention group.

## 1. Introduction

Type 2 diabetes mellitus (T2DM) is a metabolic disorder characterized by an increase in blood glucose due to impaired insulin secretion and its action [1]. The consistent hyperglycemia, insulin resistance, and insulin deficiency contribute to lipid abnormalities in T2DM [2]. The lipid abnormality is an independent risk factor for cardiovascular disease (CVD) development and commonly found in T2DM individuals [3]. Diabetic dyslipidemia is significantly associated with mortality and morbidity due to cardiovascular complications [4]. It accounts for 80% of deaths in diabetic individuals due to CVD [5]. The hyperglycemia, along with lipid abnormalities, is a modifiable risk factor for CVD, and remains uncontrolled in T2DM individuals [4,6]. In spite of advancements in therapeutic strategies, there has been no significant decrease in the mortality related to CVD [7]. The majority of T2DM individuals failed to achieve all standard goals for lipid management, and, therefore, aggressive management strategies are required to lower lipid abnormalities in T2DM individuals [7,8].

Omega-3 fatty acids are a principle component of cell membranes, which serve several important physiological functions, including as signaling molecules, transporters, and modulators of gene expression [9,10]. Previous studies reported several pharmacological activities of omega-3 fatty acids such as anti-hyperlipidemic, anti-inflammatory, and vasodilatory effects [9,11,12]. They have benefited the management of numerous chronic diseases like diabetes, CVD, and autoimmune disorders [9,13,14]. Over a period of three decades, epidemiological studies also reported that omega-3 fatty acids dietary intake provides beneficial effects in cardiovascular diseases [15,16]. Although statin drug treatment lowered the CVD incidence and its associated mortality, increased triglyceride (TG) levels and residual CVD risk remains in diabetic dyslipidemic individuals despite a decrease in LDL levels [16,17]. Therefore, adjunctive therapy is needed to lower the CVD risk. This study was designed to examine the synergistic effect of omega-3 fatty acids and oral hypoglycemic drugs i.e., glibenclamide compared with glibenclamide alone and in combination with statin drug treatment.

Various animal models have been used to assess the pathogenesis of diabetes and its associated complications [18,19,20]. High fat diet with low dose streptozotocin induces insulin resistance, hyperglycemia, hyperinsulinemia, and hyperlipidemia, which are characteristics of T2DM [21,22]. With this background, we investigated the effect of omega-3 fatty acids i.e., flax and fish oil, along with glibenclamide, against diabetic dyslipidemia by using a high fat diet with low dose streptozotocin-induced diabetic rat model.

## 2. Materials and Methods 

### 2.1. Chemicals and Reagents

Flax oil capsules were procured from the Real World Nutritional Laboratory, Pune, India (Alvel-500). Fish oil capsules purchased from a local pharmacy (Merck Ltd., Pune, India) (Maxepa-500). Streptozotocin (STZ) (Sigma-AldrichSt. Louis, Missouri, USA), and glibenclamide tablets (Daonil-5 mg; Aventis Pharma, Pune, MH, India) were procured from a local pharmacy. The lard oil was purchased from the local market. The standard chow diet was procured from Nutrivet life sciences (Pune, MH, India).

### 2.2. Animals

Design of experiment, along with their procedures and techniques, was sanctioned by the Institutional Animal Ethics Committee (IAEC) of Bharati Vidyapeeth University, Pune, India. The study was approved through sanction number: (BVDUMC/2881/2016/001/001). Forty-eight male Wistar rats weighing (120–150 gm, 10 weeks old) which were received from Medical College of Bharati Vidyapeeth, Pune, India. The animals were kept in standard animal house conditions (temperature 22 ± 2 °C and 12:12 hr light and dark cycle condition with 55 ± 5% humidity, about 3 animals per cage). The high fat diet (HFD) composition is represented in Table 1.

After acclimatization, rats were randomly distributed into eight groups (*n* = 6) and treatment protocol as follows:

All groups of animals except healthy control received high fat diet (HFD) and water *ad libitum* during the experimental period. Group I (HC): healthy control; received standard chow diet for90 days; group II (HFDC): high fat diet control; group III (DC): diabetic control received low dose streptozotocin (35 mg/kg); group IV (GC): glibenclamide control treated with STZ and glibenclamide; group V (SC): statin control treated with STZ and statin; group VI (GSC): glibenclamide—statin control given glibenclamide and statin; group VII (flax oil): received flax oil and glibenclamide; group VIII (fish oil): received fish oil and glibenclamide.

The glibenclamide and statin was given at 0.25 mg/kg and 10 mg/kg body weight (b.w.)/day, p.o. respectively. All standard drug interventions were given after the development of stable hyperglycemia. The flax and fish oil were given daily at a dose 500 mg/kg body weight (b.w.), p.o. The flax and fish oil intervention was given throughout the experiment i.e., from 1st day to 90th day. However, after the confirmation of stable hyperglycemia, flax and fish oil interventions were continued with glibenclamide (0.25 mg/kg b.w./day, p.o.) till completion of the experiment.

### 2.3. Experimental Design

**Figure 1:**
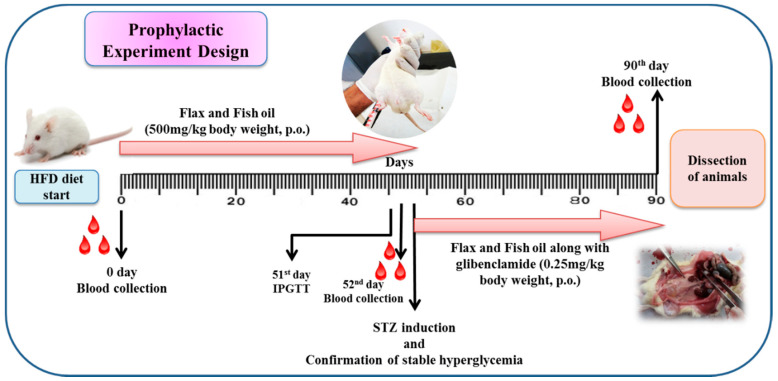
Design of the experiment. HFD: High fat diet, IPGTT: Intraperitoneal glucose tolerance test, STZ: streptozotocin; p.o. per os.

All animals were kept on a respective diet for 90 days. Intraperitoneal glucose tolerance test (IPGTT) was done on 51st day for the detection of glucose intolerance in animals. After confirmation of glucose intolerance, rats from different groups (III-VIII) were injected with a single dose of STZ (35 mg/kg body weight (b.w., i.p.) and wait for the development of stable hyperglycemia. The design of the experiment is demonstrated in Figure 1. The intake of food and water intake was recorded daily. At the end of the experiment, all animals were sacrificed. For various biochemical estimations, the blood was collected at 0 (before providing HFD), 52nd (before STZ induction) and 90th day (at end of the experiment). The different tissues like liver, kidney, pancreas, visceral adipose tissue near kidney, gastrocnemius muscle (hindlimb muscle), and heart were excised, snap-frozen immediately in liquid nitrogen and kept at −80 °C. The liver was used for gene expression studies. A small parts of the tissues (liver, pancreas, and kidney) were kept in neutral buffered formalin (10%) for the histopathological examination.

### 2.4. Intraperitoneal Glucose Tolerance Test (IPGTT)

After seven weeks of HFD, supplementation, all animal groups were fasted for 6 hrs. Initial blood glucose levels (0 min) were assessed. The glucose (2 gm/kg b.w.) solution was injected (i.p.) to all animals. Blood glucose levels were assessed using Accu-Chek monitor (Roche Diagnostics Pty. Ltd., Basel, Switzerland) at different time points (i.e., 0, 15, 30, 60, 90, and 120 min) from tail vein.

### 2.5. Assessment of Insulin Resistance

Insulin was estimated through rat-specific ELISA assay kits (Ray Biotech, GA, USA) after confirmation of glucose tolerance by IPGTT. The HOMA-IR (homeostasis model assessment of insulin resistance) was calculated as per the formula from Uma [23]:

HOMA-IR = Insulin (μU/mL) × glucose (mM)/22.5

### 2.6. Biochemical Parameters

The biochemical assessments were done by commercially available kits (Coral Clinical Systems, Goa, India). The glucose, lipid profile, liver, and kidney function markers were estimated from serum at 0 day (before providing HFD), 52nd (before STZ induction) and 90th day. Triglycerides (TGs), total cholesterol (TC), very low density lipoprotein (VLDL), low-density lipoprotein (LDL), and high density lipoprotein (HDL) were measured. In the liver function tests, serum glutamic oxaloacetic transaminase (SGOT) and serum glutamic pyruvic transaminase (SGPT) were also assessed. Urea and creatinine markers were estimated to assess the kidney function.

### 2.7. Selection of Gene for Quantitative Real-Time Polymerase Chain Reaction (qRT-PCR) Analysis

In this study, 3 transcription factors [Sterol Regulatory Element-Binding Protein-1c (SREBP-1c), Nuclear Factor-κβ (NFκβ) and Peroxisome Proliferator-Activated Receptor Gamma (PPAR-γ)] were selected, which regulates the expression of target genes, such as Fatty Acid Synthase (FASN), Acetyl-CoA Carboxylase Alpha (ACACA), Carnitine Palmitoyltransferase 1 (CPT1) and inflammatory marker [Tumor Necrosis Factor—Alpha (TNF-α)]. The Fatty Acid Binding Proteins (FABP) gene was also studied. KicqStart^®^ Primers were procured from Sigma Aldrich (New York, USA). The selected genes and their primer sequences are depicted in Table 2.

### 2.8. Assessment of Hepatic Gene Expression by qRT-PCR

Total RNA was extracted from liver by TRIZOL method (Invitrogen, Carlsbad, CA, USA). The quality of RNA was assessed by agarose gel electrophoresis (BioRad, Hercules, CA, USA). The RNA quantification was achieved by ND-1000 UV spectrophotometer (Nanodrop Technologies, Wilmington, DE, USA). For qRT-PCR assessment, the isolated RNA (2 µg) was used to synthesize cDNA by using SuperScriptTM first strand synthesis kit (Invitrogen).

The Real-time PCR analysis was done by SYBr green assays (Applied Biosystems, Waltham, Massachusetts, CA, USA) on StepOne real-time PCR system (Applied Biosystems, Waltham, Massachusetts, CA, USA). The following qRT-PCR protocol was used: initial denaturation step was done at 95 °C for 10 min. This step was followed by the 40 cycles of denaturation (95 °C for 3 s); annealing (60 °C for 30 s) and extension (95 °C for 15 s). The final extension step was achieved at 60 °C for 15 s. Three biological replicates were analyzed from each group. The reaction was carried out in duplicate and the Ct (cycle threshold) values of all samples were normalized by using Glyceraldehyde-3-Phosphate Dehydrogenase (GAPDH) (endogenous housekeeping control).

### 2.9. Histological Examination

All animals were sacrificed and different tissues (liver, pancreas, kidney, adipose tissue, muscle and heart) were collected for further analysis. The part of liver, pancreas, and kidney were fixed in buffered formalin solution (10%, pH 7). Then all tissues were embedded in paraffin for block preparation. The tissue sections were cut to 4 μm thickness and stained by Hematoxylin and Eosin. The slides were observed under the light microscope (EVOS™ FL Auto 2 Imaging System, Invitrogen, Carlsbad, CA, USA).

### 2.10. Statistical Analysis

The data are represented as Mean ± standard error (SE). Statistical analysis was carried out by using one-way analysis of variance (ANOVA) followed by Dunnett’s Multiple Comparison Test using GraphPad Instat (Version 5, GraphPad Software Inc., San Diego, CA, USA).

## 3. Results

### 3.1. Assessment of Average Body Weight, Feed and Water Consumption

The feed and water intake and body weight of experimental groups is represented in Table 3. The water and feed intake increased (*p* ≤ 0.001) in the diabetic rats (DC) as compared to the healthy control rats (HC). Initially, the average body weight of all animals was between 120–150 gm. The body weight was not statistically significant between diabetic and healthy rats. The flax oil-treated rats showed an increase (*p* ≤ 0.001) in feed consumption as compared to the diabetic rats. The omega-3 fatty acid intervention groups showed decreased (*p* ≤ 0.001) water intake as compared to the diabetic group. Whereas, body weight was significantly (*p* ≤ 0.001) elevated in flax and fish oil treatment groups as compared to the diabetic group.

### 3.2. Estimation of Organ Weight

Organ weight of all experimental animals are shown in Table 4. Diabetic rats showed increased liver, adipose tissue and muscle tissue weight (*p* < 0.001) increased as compared to the HC rats. Whereas, kidney (*p* < 0.001) and heart weight was decreased in the DC group as compared to the healthy group.

The flax (*p* < 0.001) and fish (*p* < 0.01) oil intervention group showed decreased liver weight as compared to the diabetic group. Similarly, adipose tissue weight also decreased (*p* < 0.001) in flax and fish oil intervention groups as compared to DC group. Whereas, muscle weight was decreased in both flax and fish (*p* < 0.001) oil treatment groups as compared to DC group. The flax and fish oil intervention groups showed increase in kidney weight (*p* < 0.001) as compared to DC group. The heart weight was decreased in flax oil treatment group and elevated (*p* < 0.001) in fish oil treatment group as compared to the DC.

### 3.3. Assessment of Biochemical Parameters at Zero Day

Biochemical estimations of all experimental animals before providing HFD are shown in Table 5. Serum glucose, lipid profile (total cholesterol, triglycerides, VLDL, LDL and HDL) liver (SGOT and SGPT) and kidney function tests (creatinine and urea) were found not significantly different among all experimental groups before providing of respective diet.

### 3.4. IPGTT and Area under the Curve (AUC) for the Experimental Groups

**Figure 2:**
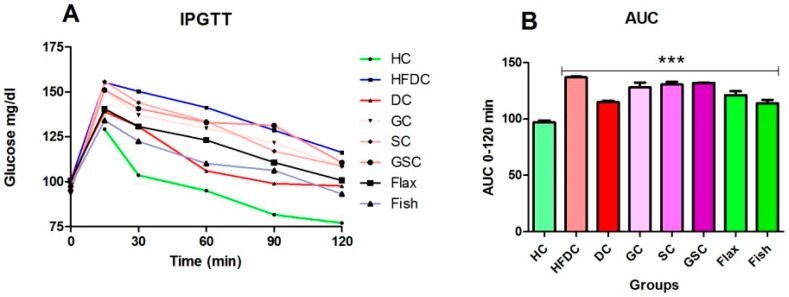
IPGTT and area under the curve (AUC) for the experimental groups. (**A**) Variations in blood glucose levels during IPGTT; (**B**) Area under the curve (AUC) for IPGTT. Results are represented as Mean ± SE (*n* = 6 for each group). *** *p* ≤ 0.001, when compared with the HC animals (Dunnett’s Multiple Comparisons Test). HC: Healthy control, HFDC: High fat diet control, DC: Diabetes control, GC: Glibenclamide control, SC: Statin control, GSC: Glibenclamide statin control.

Figure 2A,B depicts the glucose clearance and area under the curve (AUC) of IPGTT. The blood glucose levels at 0 and 120 min was elevated in all the experimental group as compared to the healthy group (Figure 2A). All experimental groups showed increase (*p* ≤ 0.001) in AUC as compared to the healthy control group. The glucose clearance was not statistically significant between HFDC, flax, and fish oil intervention groups.

### 3.5. Insulin Resistance

**Figure 3:**
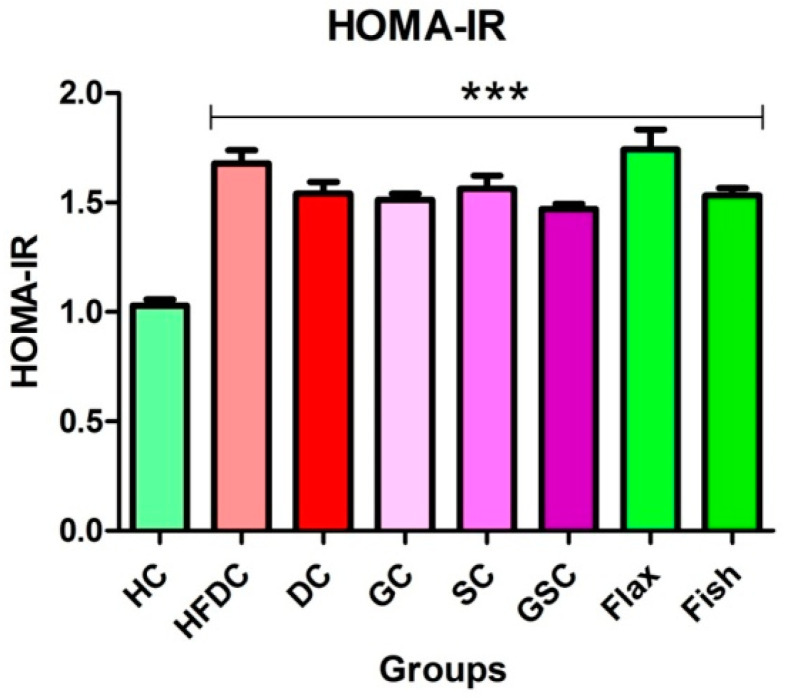
HOMA-IR of different experimental groups Results are represented as Mean ± SE (*n* = 6 for each group). *** *p* ≤ 0.001, when compared with the HC (Dunnett’s Multiple Comparisons Test). HC: Healthy control, HFDC: High fat diet control, DC: Diabetes control, GC: Glibenclamide control, SC: Statin control, GSC: Glibenclamide statin control, HOMA-IR: Homeostasis model assessment of insulin resistance.

Serum glucose and insulin levels are shown in Table 6. Serum glucose level was significantly elevated (*p* ≤ 0.001) in HFD fed rats (all groups except HC) as compared to healthy control rats. Flax and fish oil-treated rats showed non-significant difference in serum insulin level as compared to the healthy rats. HOMA-IR of all the experimental groups is represented in Figure 3. All animals from HFDC, DC and treatment groups of (GC, SC, GSC, flax and fish oil groups) rats showed a significantly increased (*p* ≤ 0.001) HOMA-IR as compared to the healthy control group. Prophylactically, omega-3 fatty acids significantly lowered lipid profile, liver function markers (SGOT and SGPT), and kidney function markers (creatinine and urea) (supplementary Figures S1 and S2).

### 3.6. Estimation of Biochemical Parameters

#### 3.6.1. Fish Oil Treatment Significantly Lowered Serum Glucose

**Figure 4:**
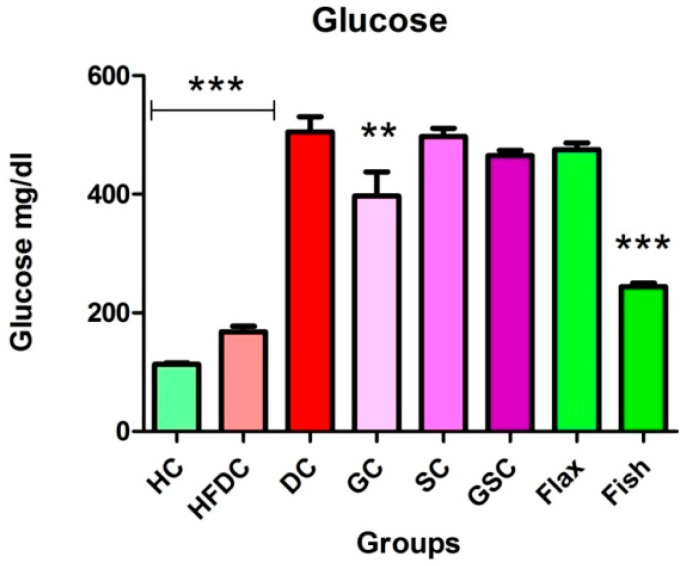
Fish oil intervention lowered serum glucose Results are represented as Mean ± SE (*n* = 6 for each group). ** *p* ≤ 0.01 and *** *p* ≤ 0.001, when compared with the DC group (Dunnett’s Multiple Comparisons Test). HC: Healthy control, HFDC: High fat diet control, DC: Diabetes control, GC: Glibenclamide control, SC: Statin control, GSC: Glibenclamide statin control.

Figure 4 represents the serum glucose levels of all the experimental groups. Serum glucose level was significantly elevated (*p* ≤ 0.001) in diabetic rats as compared to healthy control rats. Fish oil-treated rats had significantly lower (*p* ≤ 0.001) serum glucose levels as compared to the diabetic rats.

#### 3.6.2. Fish Oil Treatment Lowered Abnormal Lipid Profile

**Figure 5:**
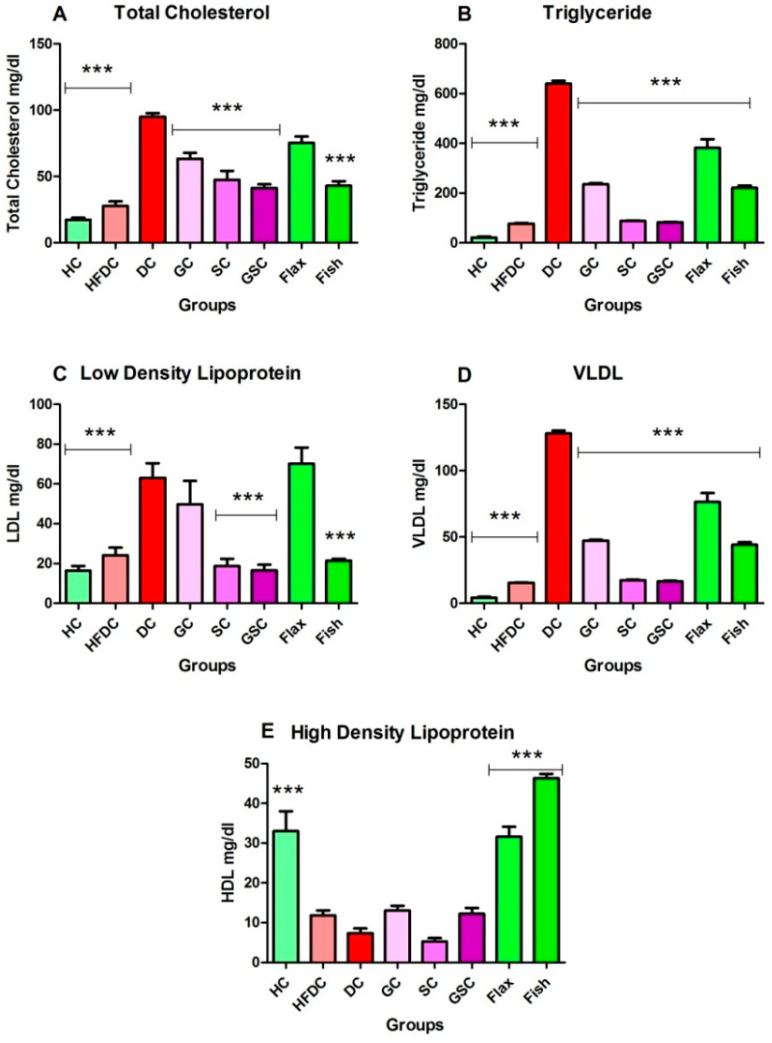
Assessment of lipid profile from experimental groups. Results are represented as Mean ± SE (*n* = 6 for each group). *** *p* ≤ 0.001, when compared with the DC group (Dunnett’s Multiple Comparisons Test). (**A**) Serum total cholesterol level, (**B**) Serum triglycerides level, (**C**) Serum low-density lipoprotein level, (**D**) Serum very low-density lipoprotein level, (**E**) Serum high-density lipoprotein level. HC: Healthy control, HFDC: High fat diet control, DC: Diabetes control, GC: Glibenclamide control, SC: Statin control, GSC: Glibenclamide statin control, LDL: Low-density lipoprotein, VLDL: Very low-density lipoprotein, HDL: High-density lipoprotein,.

Figure 5A–E depicts the serum lipid profile of all experimental groups. Diabetic animals showed significantly (*p* ≤ 0.001) increased serum TC, TGs, LDL, and VLDL levels as compared to the healthy control animals. The HDL level was significantly (*p* ≤ 0.001) decreased in diabetic animals as compared to healthy control animals. Flax and fish (*p* ≤ 0.001) oil treatment group showed decreased serum TC level as compared with diabetic group. Serum TG and VLDL levels were significantly (*p* ≤ 0.001) decreased in flax and fish oil-treated animals as compared to the diabetic animals. Serum LDL level was decreased (*p* ≤ 0.001) in fish oil-treated animals as compared to the diabetic animals. Flax and fish oil intervention elevated (*p* ≤ 0.001) serum HDL levels as compared to the DC group. Flax oil-treated animals showed significant (*p* ≤ 0.001) increase in TC, TGs, LDL, VLDL and HDL as compared to SC and GSC-treated animals. The serum TC level found to be comparable among SC, GSC and fish oil-treated groups. Serum LDL level increased in fish oil group as compared to SC and GSC group. Serum TGs, VLDL and HDL levels were significantly increased (*p* ≤ 0.001) in fish oil intervention group as compared to SC and GSC group. The fish oil treatment showed a significant decrease in abnormal lipid profile and increase serum HDL.

#### 3.6.3. Flax and Fish Oil Interventions Decreases Level of Hepatic Enzymes

**Figure 6:**
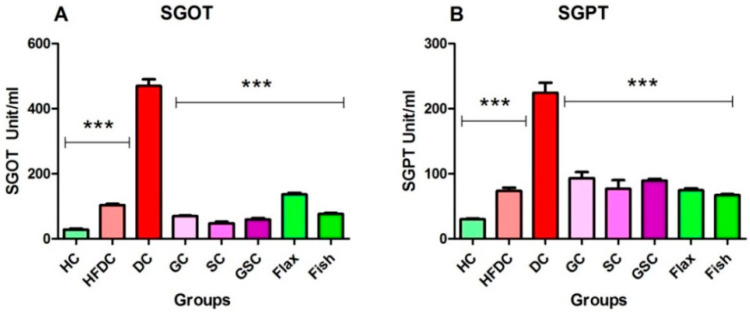
Flax and fish oil interventions lowered level of hepatic enzymes. Results are represented as Mean ± SE (*n* = 6 for each group). *** *p* ≤ 0.001, when compared with the DC group (Dunnett’s Multiple Comparisons Test). (**A**) Serum glutamic oxaloacetic transaminase level (**B**) Serum glutamic pyruvic transaminase level. HC: Healthy control, HFDC: High fat diet control, DC: Diabetes control, GC: Glibenclamide control, SC: Statin control, GSC: Glibenclamide statin control, SGOT: Serum glutamic oxaloacetic transaminase, SGPT: Serum glutamic pyruvic transaminase.

In diabetic rats, serum SGOT and SGPT levels were elevated (*p* ≤ 0.001) as compared to the healthy rats. Flax and fish oil-treated group showed significant decrease (*p* ≤ 0.001) in serum SGOT and SGPT level as compared to the diabetic group. The SGOT level was increased in flax (*p* ≤ 0.001) and fish oil intervention groups as compared to SC and GSC. Serum SGPT level decreased in both flax and fish oil groups as compared to SC and GSC. Serum SGOT and SGPT levels of all the experimental groups are represented in Figure 6A,B.

#### 3.6.4. Flax and Fish Oil Intervention Improved Kidney Function

**Figure 7:**
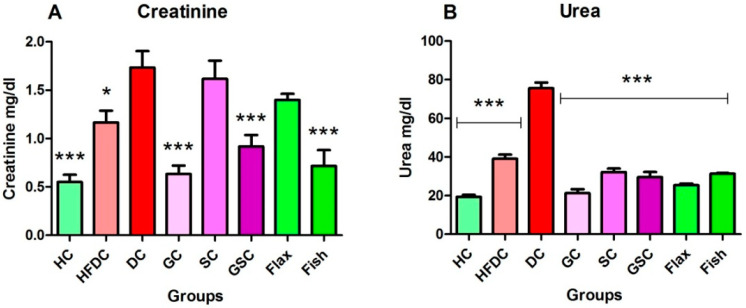
Flax and fish oil interventions improved kidney function. Results are represented as Mean ± SE (*n* = 6 for each group). * *p* ≤ 0.05 and *** *p* ≤ 0.001, when compared with the DC group (Dunnett’s Multiple Comparisons Test). (**A**) Serum creatinine level, (**B**) Serum urea level. HC: Healthy control, HFDC: High fat diet control, DC: Diabetes control, GC: Glibenclamide control, SC: Statin control, GSC: Glibenclamide statin control.

Serum creatinine and urea levels of all experimental groups were depicted in Figure 7A,B. Diabetic animals showed significantly (*p* ≤ 0.001) increased serum creatinine and urea levels as compared to the healthy control animals. Flax oil intervention groups showed decreased serum creatinine and urea (*p* ≤ 0.001) levels as compared to the diabetic group. While fish oil intervention significantly (*p* ≤ 0.001) lowered both serum creatinine and urea levels as compared to the diabetic group. Serum creatinine and urea levels were non-significantly decreased in flax oil intervention groups as compared to SC and GSC. The serum creatinine level was decreased in fish oil intervention groups as compared to SC (*p* ≤ 0.001) and GSC. Serum urea was not significantly different among SC, GSC, and fish oil groups. Fish oil intervention effectively improved kidney function.

### 3.7. Expression of Transcription Factors and Their Regulatory Genes

**Figure 8:**
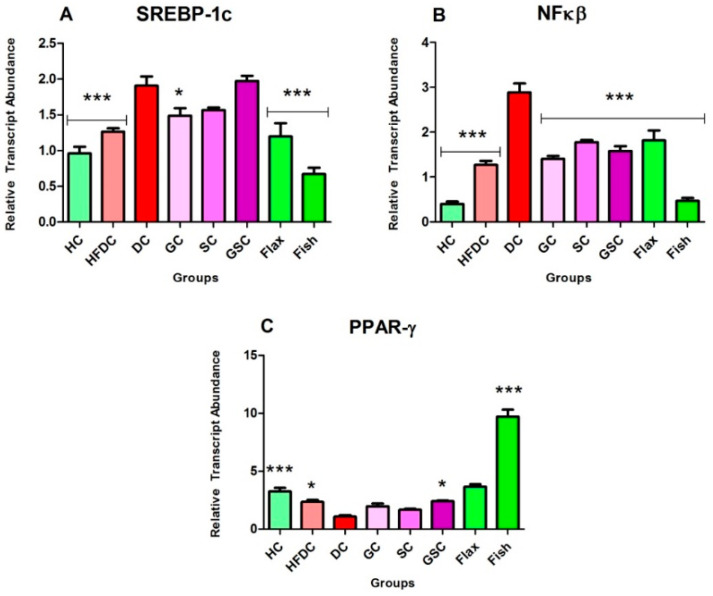
Expression of transcription factors are modulated after intervention of flax and fish oil. Results are represented as Mean ± SE (*n* = 3 for each group). * *p* ≤ 0.05 and *** *p* ≤ 0.001, when compared with the DC group (Dunnett’s Multiple Comparisons Test). (**A**) Expression of sterol regulatory element-binding proteins-1c gene, (**B**) Expression of nuclear factor-κβ gene, (**C**) Expression of peroxisome proliferator-activated receptor gamma gene. HC: Healthy control, HFDC: High fat diet control, DC: Diabetic control, PPAR-γ: Peroxisome proliferator-activated receptor gamma, SREBP-1c: Sterol regulatory element-binding proteins-1c, NFκβ: Nuclear factor-κβ.

**Figure 9:**
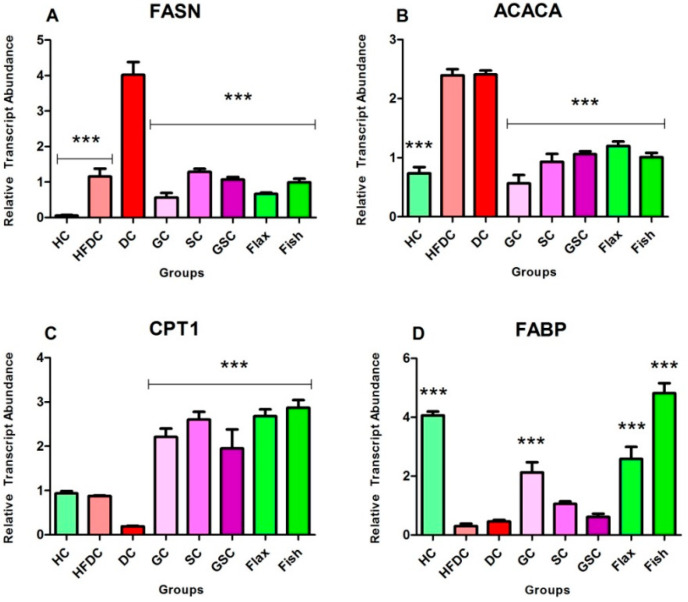
Expression profiles of fatty acid metabolism genes. Results are denoted as Mean ± SE (*n* = 3 for each group). *** *p* ≤ 0.001, when compared with the DC group (Dunnett’s Multiple Comparisons Test). (**A**) Expression of fatty acid synthase gene, (**B**) Expression of acetyl-CoA carboxylase alpha gene, (**C**) Expression of carnitine palmitoyl transferase 1 gene, (**D**) Expression of fatty-acid-binding proteins gene. HC: Healthy control, HFDC: High fat diet control, DC: Diabetic control, ACACA: Acetyl-CoA carboxylase alpha, FASN: Fatty acid synthase, CPT1: Carnitine palmitoyl transferase 1, FABP: Fatty-acid-binding proteins.

**Figure 10:**
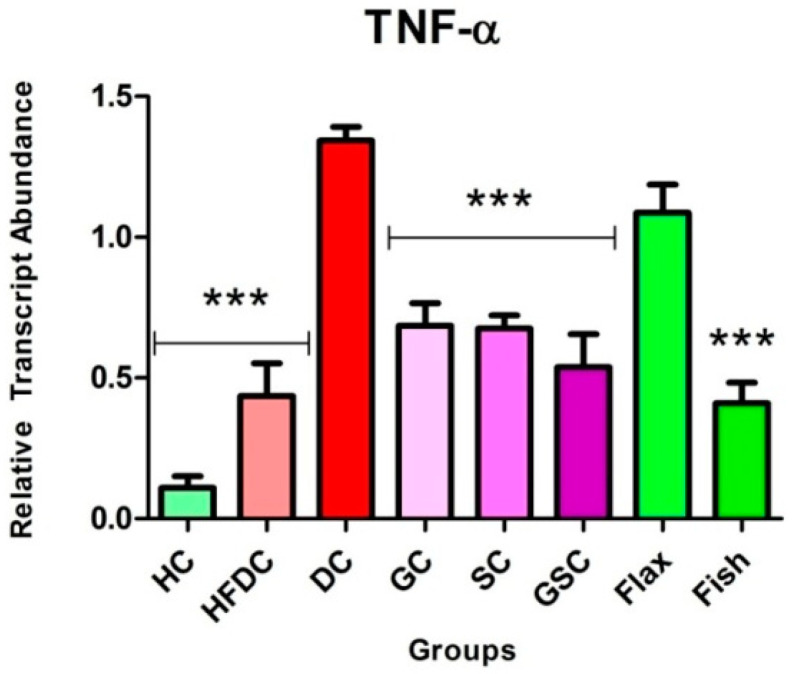
Expression of TNF-α in experimental groups. Results are represented as Mean ± SE (*n* = 3 for each group). *** *p* ≤ 0.001, when compared with the DC group (Dunnett’s Multiple Comparisons Test). HC: Healthy control, HFDC: High fat diet control, DC: Diabetic control group-treated STZ and TNF-α: Tumor necrosis factor-alpha.

In the present study, we have examined the effect of flax and fish oil along with glibenclamide against diabetic dyslipidemia. For gene expression studies, three transcription factors and five regulatory genes were examined. The expression profiles are shown in (Figure 8, Figure 9 and Figure 10). qRT-PCR amplification efficiencies are depicted in Table 7.

#### 3.7.1. Flax and Fish Oil Interventions Modulates the Expression of Transcription Factors Resulting in Lipid Normalization

The expression of transcription factors is depicted in Figure 8A–C. In diabetic animals (DC), SREBP-1c expression was significantly (*p* ≤ 0.001) upregulated by ~1.98 and ~1.51-fold as compared to the HC and HFDC animals, respectively. Comparatively, flax and fish oil intervention groups showed significant downregulation by ~1.59 and ~2.84-fold as compared to the diabetic group.

NFκβ gene expression was significantly (*p* ≤ 0.001) upregulated in the diabetic animals by ~7.27 and ~2.27-fold as compared to the HC and HFDC animals, respectively. On the other hand, its expression was significantly (*p* ≤ 0.001) downregulated by ~1.59 and ~6.16-fold in flax and fish oil treatment groups as compared with the diabetic group.

Expression of PPAR-γ was significantly downregulated in diabetic animals by ~3.00 and ~2.15-fold, as compared to the HC (*p* ≤ 0.001) and HFDC (*p* ≤ 0.05) control animals. Flax oil-treated animals showed non-significant increased expression as compared to the diabetic group. While, fish oil-treated animals showed significant (*p* ≤ 0.001) upregulation expression by ~8.95, ~4.94, ~5.84, and ~4.04-fold as compared to the diabetic animals.

#### 3.7.2. Flax and Fish oil Intervention Modulates the Expression Fatty Acid Metabolism Genes which Results in Decreased Lipid Abnormality

Figure 9A–D represents the expression of lipid metabolism genes. Expression of FASN genes was significantly upregulated (*p* ≤ 0.001) in the diabetic animals by ~70.48 and ~3.47-fold as compared to HC and HFDC animals. Flax and fish oil-treated animals showed downregulation (*p* ≤ 0.001) by ~6.01 and ~4.04-fold as compared to the diabetic animals.

ACACA gene expression was significantly (*p* ≤ 0.001) upregulated in the diabetic rats by ~3.26-fold as compared to the healthy rats. Flax (*p* ≤ 0.001) and fish (*p* ≤ 0.05) oil-treated groups showed upregulation by ~2.12 and ~1.78-fold as compared to the diabetic group, respectively.

Expression of CPT1 genes was non-significantly downregulated in the diabetic rats by ~4.95 and ~4.63-fold as compared to the HC and HFDC rats. Flax and fish oil intervention groups showed significant upregulation (*p* ≤ 0.001) by ~14.17 and ~15.20-fold as compared to the diabetic group.

FABP gene expression was significantly downregulated (*p* ≤ 0.001) by ~8.87-fold in diabetic rats as compared to the healthy rat group. Both flax and fish oil intervention groups showed significant upregulation (*p* ≤ 0.001) by ~5.64 and ~10.51-fold as compared to the diabetic groups, respectively.

#### 3.7.3. Fish Oil Intervention Downregulates the Expression of TNF-α

In DC rats, TNF-α gene expression was upregulated (*p* ≤ 0.001) by ~12.31 and ~3.09-fold as compared to the healthy and high fat diet control rats, respectively. Comparatively, flax and fish (*p* ≤ 0.001) oil-treated groups showed downregulation by ~1.24 and ~3.28-fold as compared to the diabetic group, respectively. Figure 10 represents the inflammatory gene expression, TNF-α.

### 3.8. Histological Examination of Liver, Pancreas, and Kidney From Experimental Animals

**Figure 11:**
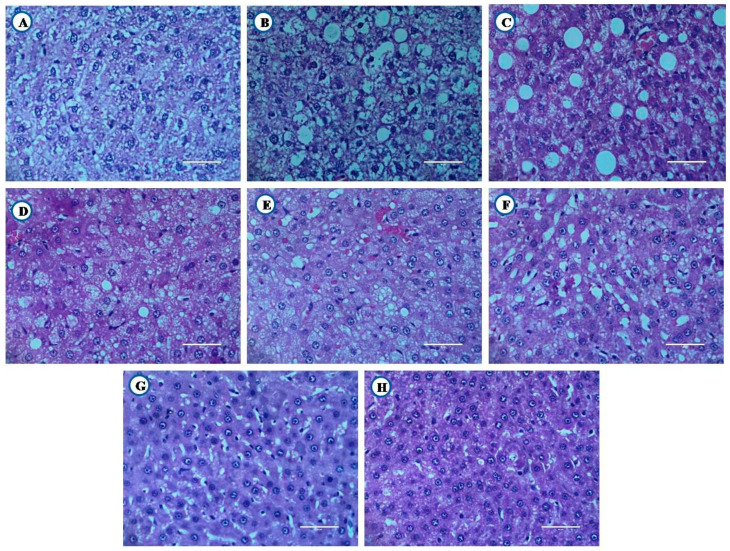
Histological examination of liver tissue (Scale bar 50 µm). Healthy. (**A**) Healthy control, (**B**) High fat diet control, (**C**) Diabetic control, (**D**) Glibenclamide control, (**E**) Statin control, (**F**) Glibenclamide statin control, (**G**) Flax oil (500 mg/kg), (**H**) Flax oil (500 mg/kg).

**Figure 12:**
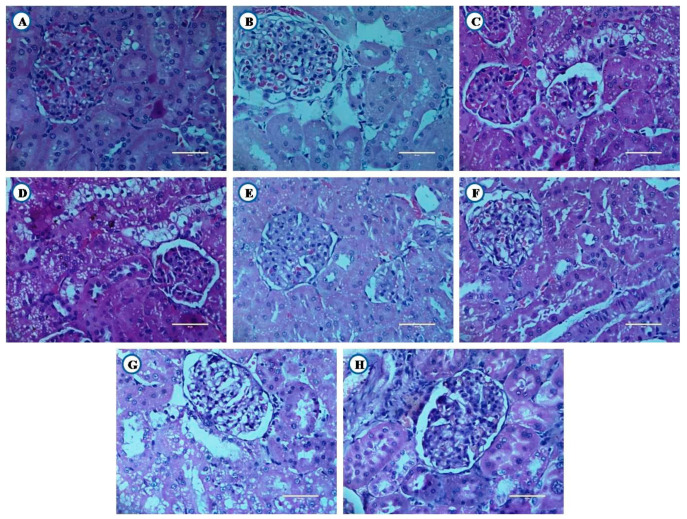
Histological examination of kidney (Scale bar 50 µm). (**A**) Healthy control, (**B**) High fat diet control, (**C**) Diabetic control, (**D**) Glibenclamide control, (**E**) Statin control, (**F**) Glibenclamide statin control, (**G**) Flax oil (500 mg/kg), (**H**) Flax oil (500 mg/kg).

**Figure 13:**
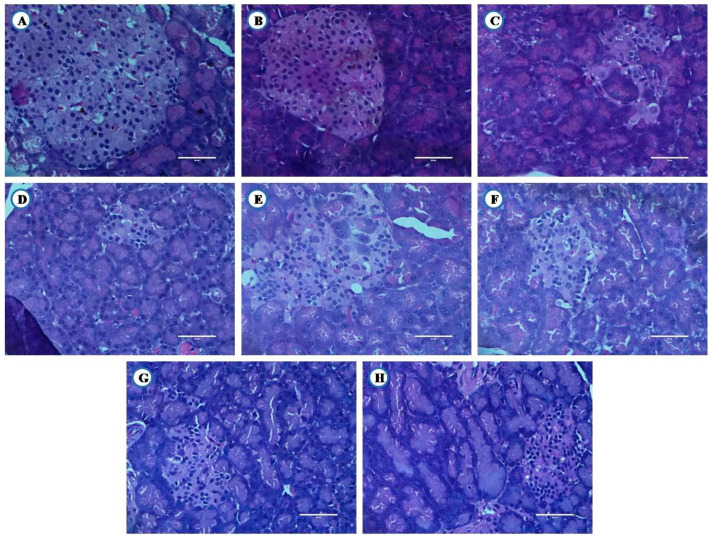
Histological examination of pancreas (Scale bar 50 µm). (**A**) Healthy control, (**B**) High fat diet control, (**C**) Diabetic control, (**D**) Glibenclamide control, (**E**) Statin control, (**F**) Glibenclamide statin control, (**G**) Flax oil (500 mg/kg), (**H**) Flax oil (500 mg/kg).

Animals from experimental groups developed typical changes in liver, pancreas and kidney. Their histopathological examination is shown in Figure 11, Figure 12 and Figure 13.

#### 3.8.1. Histological Examination of Liver

Healthy animals showed normal architecture of hepatocytes (Figure 11A). High fat diet control group rats showed focal fatty changes in the liver (Figure 11B). Diabetic animals develop microvesicular fatty changes in the liver (Figure 11C). The flax oil intervention, along with glibenclamide showed focal fatty changes in the liver (Figure 11G). However, fish oil intervention, along with glibenclamide showed near-normal architecture of hepatocytes (Figure 11H).

#### 3.8.2. Histological Examination of Kidney

Healthy and high fat diet control group animals showed normal architecture of the kidney (Figure 12A,B). Diabetic rats showed tubules with vacuolated cells (Figure 12C). The animals receiving standard drugs (GC, SC and GSC) also showed tubules with vacuolated cells (Figure 12D,F). Flax and fish oil interventions along with the combination of glibenclamide showed tubules with vacuolated cells (Figure 12G,H).

#### 3.8.3. Histological Examination of Pancreas

Healthy and high fat diet control animals showed normal architecture of the pancreatic tissue (Figure 13A,B). Diabetic rats showed reduced number and size of islets of Langerhans and β cells (Figure 13C). Flax and fish oil intervention group showed reduced number and size of Langerhans and β cells (Figure 13G,H).

## 4. Discussion

The provision of HFD, along with a low dose of streptozotocin in rats, results in a condition that mimics the pathophysiology of type 2 diabetes (T2DM) in humans and is thus a suitable model for the practical investigations and testing of different natural compounds for the effective management of type 2 diabetes and its complications [20,21,22]. Despite advancement in the prevention and management strategies of diabetes and its associated complications in recent years, still, it has been growing alarmingly with the high rate of morbidity and mortality [24]. Therefore, aggressive management strategies for T2DM and its associated lipid abnormalities are highly recommended [4,22].

STZ-treated diabetic rats showed decreased body weights and elevated blood glucose, which are characteristic features of diabetes [18]. In the present study, a significant decrease in body weight and sustained hyperglycemia was observed in diabetic rats. The lipid abnormality is a very frequent impairment in type 2 diabetes patients [24]. For its management, they are commonly prescribed lipid-lowering drugs like statins. Therefore, one of the groups was given treatment with a normolipidemic drug just to evaluate the effect of the same drug on lipid abnormalities.

Several studies have reported the triglyceride-lowering effect of omega-3 fatty acids [9,25,26,27]. Some also studied cardioprotective effects of omega-3 fatty acids in animal models as well as in humans [28]. Flax oil, is a major source of alpha linolenic acid (ALA), and fish oil, predominantly contain eicosapentaenoic acid (EPA) and docosahexaenoic acid (DHA) are the principle sources of omega-3 fatty acids [9]. Previous studies showed that, flax and fish oil treatment lowered abnormal lipid profile in diabetic rats [9]. Our results are in accordance with previous findings. Both flax and fish oil exhibit beneficial effects on hepatic cholesterol metabolism in HFD-fed animals [29].

Hendrich [30] reviewed the effect of omega-3 fatty acid in human clinical trials and concluded that its effects in T2DM were not well studied [9,30]. The management of metabolic disorders (T2DM and its associated complications) recommended combining lifestyle changes with pharmacological therapy [31]. In this regard, several studies reported, the beneficial effect of omega-3 fatty acids with different allopathic drugs (thiazolidinediones, pioglitazone, rosiglitazone, etc.) in HFD-fed mice results in the increased adiponectin secretion [31,32]. With this background, we have studied the effect of flax and fish oil intervention in combination with glibenclamide in HFD with low STZ-induced diabetic dyslipidemia. The present study helps to fill the gap and investigates the combinational effect of omega-3 fatty acids and oral hypoglycemic drug on lipid abnormalities through modulation of transcription factors and their regulatory genes.

**Figure 14:**
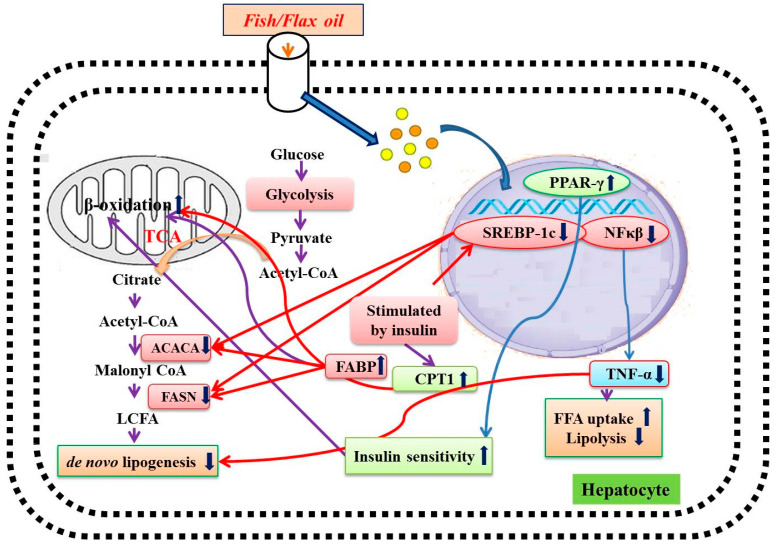
Mechanism of action of flax and fish oil intervention. PPAR-γ: Peroxisome proliferator-activated receptor gamma, SREBP-1c: Sterol regulatory element-binding proteins-1c, NFκβ: Nuclear factor-κβ, ACACA: Acetyl-CoA carboxylase alpha, FASN: Fatty acid synthase, CPT1: Carnitine palmitoyl transferase 1, FABP: Fatty-acid-binding proteins, TNF-α: Tumor necrosis factor-alpha, TCA: Tricarboxylic acid cycle, FFA: Free fatty acids, LCFA: Long chain fatty acids.

Several studies reported that flax and fish oil intervention exhibited triglyceride-lowering effect in streptozotocin-induced diabetic rats [9,27,33,34]. However, the treatment did not show any effect on serum TC and LDL levels. In the present study, flax and fish oil along with glibenclamide treatment effectively lowered serum TC, triglycerides, VLDL, and LDL levels in diabetic rats. It has been previously reported that flax and fish oil intervention significantly increase HDL levels in STZ-induced diabetic rats [9,27,33]. In our study, a similar trend was observed. Thus, effective lowering of the abnormal lipid profile was observed in fish oil and glibenclamide combinational treatment. The overall mechanism of the action of flax and fish oil on hepatic gene expression is depicted in Figure 14.

SREBP is an important transcription factor that plays a crucial role in the regulation of fatty acid and cholesterol metabolism in the liver [35]. It consists of two isoforms i.e., SREBP-1a and SREBP-1c, which are expressed highly in the liver. Its overexpression was associated with elevated levels of cholesterol and triglycerides [36,37]. Earlier studies document that upregulated lipogenic gene expression, such as for fatty acid synthase (FASN) and acetyl-CoA carboxylase (ACACA), was resulting in increased SREBP-1c expression, which leads to hepatic steatosis [38,39]. Hence, downregulation of SREBP-1c has a therapeutic value in the treatment of diabetic dyslipidemia [22,40,41]. The omega-3 fatty acid supplementation effectively lowered triglycerides level through downregulation of SREBP-1c gene expression [9,27,42]. Similarly, in our study, both flax and fish oil-treated animals showed downregulated SREBP-1c expression followed by a decrease in the expression of FASN and ACACA genes. These gene modulations may be one of the reasons for lowering the lipid abnormalities in flax and fish oil-treated rats.

CPT1 gene plays a major role in the uptake of fatty acids by the mitochondria for fatty acids β-oxidation [43]. In our study, the CPT1 expression was found to be increased in flax and fish oil-treated animals. This might be the probable reason for lowering serum triglyceride levels in diabetic rats.

Several studies reported that NF-κβ, a transcription factor (TF), plays a crucial role in insulin resistance and T2DM pathogenesis [22,41,44,45]. In diabetic conditions, upregulated NF-κβ expression leads to an increase inflammatory cytokine expression, e.g. tumor necrosis factor-α (TNF-α) [22,46,47]. In turn, it is associated with atherosclerotic lesions, lipolysis, and lipogenesis. Overall, this may result in an increased risk of cardiovascular complications in T2DM individuals [46,47]. In the present study, both TNF-α expression and its transcription factor NF-κβ were found to be downregulated in flax and fish oil-treated groups.

PPAR-γ, a transcription factor, is a member of the nuclear receptor family PPARs [48]. It plays an important role in carbohydrate and lipid homeostasis [48]. The activation of PPAR-γ stimulates β-oxidation of fatty acids and it results in lower serum triglyceride levels [49]. In the present study, flax and fish oil treatment upregulated the expression of PPAR-γ, and this may result in decreased serum triglyceride levels.

The FABP are members of a multigene family of cytoplasmic lipid transport proteins [50]. It is a potential target in the treatment of insulin resistance, lipid abnormalities, and atherosclerosis [50]. It facilitates fatty acid oxidation in the liver and may be beneficial for normalizing the hyperlipidemic condition [51]. Newberry et al. [52] reported that the L-FABP-null mice exhibit poor triglyceride accumulation in the liver, which leads to an increased serum triglyceride level [52]. A Wolfrum et al. [53] study shows that L-FABP acts as a gateway for the hypolipidemic drug and polyunsaturated fatty acids, which acts as a PPAR agonists [53]. Thus, upregulation of L-FABP expression would enhance the activation of PPAR through these agonists. In the present study, both flax and fish oil supplementation upregulated the expression of L-FABP in diabetic rats and this might be one of the reasons behind lowering serum triglyceride levels. FABPs are also associated with the docosahexaenoic acid (DHA) uptake and this might be the reason behind accelerating β-oxidation of fatty acids through higher activation of PPAR. This ultimately results in lowering serum triglyceride levels [54]. Our results are in accordance with the above findings [53,54].

## 5. Conclusions

The combinational treatment of glibenclamide and flax/fish oil intervention prophylactically against diabetic dyslipidemic rats exhibited potential effects on improving lipid abnormalities through modulating the expression of transcription factors (SREBP1-c, NF-kβ and PPAR-γ) and their regulatory genes i.e., ACACA, FASN, CPT1, FABP, and TNF-α. In the future, combination therapy of glibenclamide and omega-3 fatty acid intervention at a therapeutic level is worth investigation in the diabetic dyslipidemic condition.

## Figures and Tables

**Table 1:** High fat diet (HFD) composition.

Sr. No.	Ingredients	Weight (gm/Kg)
1	Powdered normal pellet diet	700
2	Lard oil	300

**Table 2:** The selected genes and their primer sequences.

Sr No	Target Genes	Primers	Sequences
**Housekeeping Gene**			
1	Glyceraldehyde-3-phosphate dehydrogenase	Forward Reverse	AGTTCAACGGCACAGTCAAG TACTCAGCACCAGCATCACC
**Transcription Factors**			
1	Sterol Regulatory Element-Binding Proteins-1c	Forward Reverse	AAACCTGAAGTGGTAGAAAC TTATCCTCAAAGGCTGGG
2	Peroxisome Proliferator-Activated Receptor Gamma	Forward Reverse	AAGACAACAGACAAATCACC CAGGGATATTTTTGGCATACTC
3	Nuclear Factor-κβ	Forward Reverse	AAAAACGAGCCTAGAGATTG ACATCCTCTTCCTTGTCTTC
**Fatty Acid Metabolism Genes**			
1	Fatty Acid Synthase	Forward Reverse	AAAAGGAAAGTAGAGTGTGC GACACATTCTGTTCACTACAG
2	Acetyl-CoA Carboxylase Alpha	Forward Reverse	AGCAGTATTTGAACACATGG CAGTTCCAAGAAGTAGAAGC
3	Carnitine Palmitoyltransferase 1	Forward Reverse	CACTGATGAAGGAAGAAGAC CCAGTCACTCACGTAATTTG
4	Fatty Acid Binding Protein	Forward Reverse	TGGAGGGTGACAATAAAATG TCATGGTATTGGTGATTGTG
**Inflammatory Marker**			
1	Tumor Necrosis Factors-α	Forward Reverse	CTCACACTCAGATCATCTTC GAGAACCTGGGAGTAGATAAG

**Table 3:** Average body weight, feed, and water intake of the experimental groups.

Groups	Weight (gm)	Feed Intake (gm)	Water Intake (mL)
HC	199 ± 2.73	102 ± 0.09 ***	209.1 ± 1.1 ***
HFDC	279 ± 4.66 ***	181.8 ± 0.79 ***	136.4 ± 0.9 ***
DC	199 ± 2.73	110.2 ± 0.36	347.4 ± 0.3
GC	286 ± 9.11 ***	162.1 ± 1.53 ***	141.6 ± 0.5 ***
SC	275 ± 3.62 ***	143.9 ± 0.59 ***	139.8 ± 1.1 ***
GSC	267 ± 3.35 ***	169.9 ± 1.22 ***	133 ± 0.4 ***
Flax	253 ± 4.96 ***	114.9 ± 0.28 ***	144.5 ± 0.6 ***
Fish	315 ± 12.15 ***	110.6 ± 0.94	142.6 ± 0.3 ***

Results are denoted as Mean ± SE (standard error) (*n* = 6 for each group). *** *p* ≤ 0.001, when compared with the diabetic control group (Dunnett’s Multiple Comparisons Test). HC: Healthy control, HFDC: High fat diet control, DC: Diabetes control, GC: Glibenclamide control, SC: Statin control, GSC: Glibenclamide statin control.

**Table 4:** Measurement organ weight (gm).

Groups	Liver	Kidney	Adipose Tissue	Muscle	Heart
HC	8.16 ± 0.17 ***	2.14 ± 0.05 ***	3.99 ± 0.09 ***	2.51 ± 0.07 ***	1.65 ± 0.05
HFDC	12.43 ± 0.19	1.64 ± 0.08	15.17 ± 0.71	4.84 ± 0.33	1.90 ± 0.03 ***
DC	11.31 ± 1.12	1.81 ± 0.04	14.43 ± 0.74	4.94 ± 0.72	1.52 ± 0.09
GC	10.85 ± 0.34	2.67 ± 0.05 ***	4.39 ± 0.39 ***	4.12 ± 0.15	1.75 ± 0.09
SC	9.75 ± 0.36	2.27 ± 0.07 **	3.47 ± 0.15 ***	5.58 ± 0.25	1.50 ± 0.08
GSC	10.37 ± 0.28	2.28 ± 0.14 **	3.90 ± 0.46 ***	5.34 ± 0.35	1.43 ± 0.03
Flax	8.55 ± 0.19 ***	2.43 ± 0.03 ***	1.75 ± 0.04 ***	4.29 ± 0.08	1.39 ± 0.01
Fish	8.59 ± 0.05 **	2.44 ± 0.13 ***	2.88 ± 0.18 ***	2.85 ± 0.08 ***	1.90 ± 0.01 ***

Results are recorded as Mean ± SE (*n* = 6 for each group). ** *p* ≤ 0.01 and *** *p* ≤ 0.001, when compared with the diabetic control group (Dunnett’s Multiple Comparisons Test). HC: Healthy control, HFDC: High fat diet control, DC: Diabetes control, GC: Glibenclamide control, SC: Statin control, GSC: Glibenclamide statin control.

**Table 5:** Biochemical assessment before initiation of HFD.

Parameters	HC	HFDC	DC	GC	SC	GSC	Flax	Fish
Glu (mg/dl)	67.95 ± 6.05	68.33 ± 4.38	66.20 ± 9.09	61.18 ± 8.61	73.10 ± 4.34	53.97 ± 3.18	60.85 ± 2.03	56.08 ± 4.17
**Lipid profile (mg/dl)**								
TC	43.58 ± 4.37	57.63 ± 5.97	51.07 ± 3.15	49.70 ± 6.35	42.30 ± 2.63	44.48 ± 4.52	41.13 ± 4.76	47.97 ± 3.35
TGs	30.58 ± 5.71	47.40 ± 2.20	46.72 ± 2.63	36.62 ± 6.48	35.8 ± 6.63	26.47 ± 3.67	41.77 ± 6.32	33.90 ± 4.66
HDL	17.95 ± 0.20	22.19 ± 2.35	23.70 ± 1.82	23.95 ± 0.95	23.20 ± 2.50	26.83 ± 0.00	22.49 ± 0.76	21.78 ± 3.30
LDL	17.56 ± 0.10	13.37 ± 1.47	12.27 ± 2.17	16.50 ± 0.25	14.50 ± 0.66	13.80 ± 2.21	12.43 ± 0.89	12.43 ± 0.53
VLDL	6.12 ± 1.13	9.50 ± 0.44	9.31 ± 0.52	7.33 ± 1.28	7.17 ± 1.32	5.30 ± 0.74	8.36 ± 1.26	6.78 ± 0.92
**Liver Function Test (Unit/mL)**								
SGOT	41.60 ± 2.57	39.30 ± 5.09	55.87 ± 1.50	57.63 ± 4.87	45.52 ± 6.17	44.03 ± 2.05	44.68 ± 2.63	46.40 ± 5.20
SGPT	31.35 ± 3.90	34.45 ± 2.12	25.51 ± 2.53	26.60 ± 3.10	38.82 ± 2.52	34.03 ± 4.02	34.66 ± 2.89	33.75 ± 1.13
**Kidney Function Test (mg/dl)**								
Creatinine	0.60 ± 0.08	0.70 ± 0.07	0.57 ± 0.07	0.54 ± 0.07	0.55 ± 0.09	0.46 ± 0.12	0.55 ± 0.08	0.57 ± 0.06
Urea	26.49 ± 3.05	26.24 ± 0.59	25.05 ± 3.82	29.22 ± 1.04	27.55 ± 3.25	30.48 ± 0.45	28.19 ± 1.07	22.53 ± 1.53

Results are represented as Mean ± SE (*n* = 6 for each group and reactions were carried out in triplicates). All values for experimental groups were non-significantly different as compared with the healthy control group (Dunnett’s Multiple Comparisons Test). Glu: Glucose, TC: Total cholesterol, TGs: Triglycerides, LDL: Low-density lipoprotein, VLDL: Very low-density lipoprotein, HDL: High-density lipoprotein, SGOT: Serum glutamic oxaloacetic transaminase, SGPT: Serum glutamic pyruvic transaminase.

**Table 6:** Serum glucose and insulin for HOMA-IR assessment.

Experimental Groups	Glucose (mM)	Insulin (μU/mL)
HC	3.682 ± 0.10	6.293 ± 0.04
HFDC	5.208 ± 0.14 ***	7.248 ± 0.11 ***
DC	4.81 ± 0.15 ***	7.21 ± 0.10 ***
GC	4.995 ± 0.08 ***	6.822 ± 0.17 *
SC	5.134 ± 0.15 ***	6.852 ± 0.17 *
GSC	4.875 ± 0.02 ***	6.788 ± 0.14 *
Flax	6.105 ± 0.30 ***	6.418 ± 0.12
Fish	5.458 ± 0.12 ***	6.315 ± 0.03

Results are represented as Mean ± SE (*n* = 6 for each group). * *p* ≤ 0.05 and *** *p* ≤ 0.001, when compared with the HC (Dunnett’s Multiple Comparisons Test). HC: Healthy control, HFDC: High fat diet control, DC: Diabetes control, GC: Glibenclamide control, SC: Statin control, GSC: Glibenclamide statin control, HOMA-IR: Homeostasis model assessment of insulin resistance.

**Table 7:** The qRT-PCR efficiency for absolute mRNA quantification.

No.	Target Genes	Symbol	Efficiency
**Housekeeping Gene**			
1	Glyceraldehyde-3-phosphate dehydrogenase	GAPDH	97.27
**Transcription Factors**			
1	Sterol Regulatory Element-Binding Proteins-1c	SREBP-1c	91.14
2	Peroxisome Proliferator-Activated Receptor Gamma	PPAR-γ	91.81
3	Nuclear Factor-κβ	NF-κβ	101.90
**Fatty Acid Metabolism Genes**			
1	Fatty Acid Synthase	FASN	91.67
2	Acetyl-CoA Carboxylase Alpha	ACACA	84.87
3	Carnitine Palmitoyltransferase 1	CPT 1	101.65
4	Fatty Acid Binding Protein	FABP	92.96
**Inflammatory Marker**			
1	Tumor Necrosis Factors-α	TNF-α	107.09

Efficiency is calculated from the slope of the curve as E = 10(−1/slope)^−1^.

## Supplementary Materials

The following are available online at https://www.mdpi.com/2072-6643/12/12/3652/s1, Figure S1: Assessment of glucose and lipid profile before diabetes development, Figure S2: LFT and KFT before diabetes development.

## References

[1] Brown T.J., Brainard J., Song F., Wang X., Abdelhamid A., Hooper L. (2019). Omega-3, omega-6, and total dietary polyunsaturated fat for prevention and treatment of type 2 diabetes mellitus: Systematic review and meta-analysis of randomised controlled trials. BMJ.

[2] Adiels M., Olofsson S.O., Taskinen M.R., Boren J. (2006). Diabetic dyslipidaemia. Curr. Opin. Lipidol..

[3] Narindrarangkura P., Bosl W., Rangsin R., Hatthachote P. (2019). Prevalence of dyslipidemia associated with complications in diabetic patients: A nationwide study in thailand. Lipids Health Dis..

[4] Krishnaswami V. (2010). Treatment of dyslipidemia in patients with type 2 diabetes. Lipids Health Dis..

[5] Mithal A., Majhi D., Shunmugavelu M., Talwarkar P.G., Vasnawala H., Raza A.S. (2014). Prevalence of dyslipidemia in adult Indian diabetic patients: A cross sectional study (SOLID). Indian J. Endocrinol. Metab..

[6] Saydah S.H., Fradkin J., Cowie C.C. (2004). Poor control of risk factors for vascular disease among adults with previously diagnosed diabetes. JAMA.

[7] Parikh R.M., Joshi S.R., Menon P.S., Shah N.S. (2010). Prevalence and pattern of diabetic dyslipidemia in Indian type 2 diabetic patients. Diabetes Metab. Syndr. Clin. Res. Rev..

[8] Goff D.C., Gerstein H.C., Ginsberg H.N., Cushman W.C., Margolis K.L., Byington R.P., Buse J.B., Genuth S., Probstfield J.L., Simons-Morton D.G. (2007). Prevention of cardiovascular disease in persons with type 2 diabetes mellitus: Current knowledge and rationale for the Action to Control Cardiovascular Risk in Diabetes (ACCORD) trial. Am. J. Cardiol..

[9] Devarshi P.P., Jangale N.M., Ghule A.E., Bodhankar S.L., Harsulkar A.M. (2013). Beneficial effects of flaxseed oil and fish oil diet are through modulation of different hepatic genes involved in lipid metabolism in streptozotocin-nicotinamide induced diabetic rats. Genes Nutr..

[10] Liu J., Ma D.W. (2014). The role of n-3 polyunsaturated fatty acids in the prevention and treatment of breast cancer. Nutrients.

[11] Jangale N.M., Devarshi P.P., Dubal A.A., Ghule A.E., Koppikar S.J., Bodhankar S.L., Chougale A.D., Kulkarni M.J., Harsulkar A.M. (2013). Dietary flaxseed oil and fish oil modulates expression of antioxidant and inflammatory genes with alleviation of protein glycation status and inflammation in liver of streptozotocin-nicotinamide induced diabetic rats. Food Chem..

[12] Connor W.E. (2000). Importance of n-3 fatty acids in health and disease. Am. J. Clin. Nutr..

[13] Simopoulos A.P. (2002). Omega-3 fatty acids in inflammation and autoimmune diseases. J. Am. Coll. Nutr..

[14] Wu J.H., Micha R., Imamura F., Pan A., Biggs M.L., Ajaz O., Djousse L., Hu F.B., Mozaffarian D. (2012). Omega-3 fatty acids and incident type 2 diabetes: A systematic review and meta-analysis. Br. J. Nutr..

[15] Bang H.O., Dyerberg J., Nielsen A.B. (1971). Plasma lipid and lipoprotein pattern in Greenlandic West-coast Eskimos. Lancet.

[16] Fialkow J. (2016). Omega-3 fatty acid formulations in cardiovascular disease: Dietary supplements are not substitutes for prescription products. Am. J. Cardiovasc. Drugs.

[17] Kim C.H., Han K.A., Yu J., Lee S.H., Jeon H.K., Kim S.H., Kim S.Y., Han K.H., Won K., Kim D.B. (2018). Efficacy and safety of adding omega-3 fatty acids in statin-treated patients with residual hypertriglyceridemia: ROMANTIC (Rosuvastatin-Omacor in residual hypertriglyceridemia), a randomized, double-blind, and placebo-controlled trial. Clin. Ther..

[18] Srinivasan K., Ramarao P. (2007). Animal models in type 2 diabetes research: An overview. Indian J. Med. Res..

[19] Zhang M., Lv X.Y., Li J., Xu Z.G., Chen L. (2008). The characterization of high-fat diet and multiple low-dose streptozotocin induced type 2 diabetes rat model. Exp. Diabetes Res..

[20] Binh D.V., Dung N.T.K., Thao L.T.B., Nhi N.B., Chi P.V. (2013). Macro- and microvascular complications of diabetes induced by high-fat diet and low-dose streptozotocin injection in rats model. Int. J. Diabetes Res..

[21] Srinivasan K., Viswanad B., Asrat L., Kaul C.L., Ramarao P. (2005). Combination of high-fat diet-fed and low-dose streptozotocin-treated rat: A model for type 2 diabetes and pharmacological screening. Pharmacol. Res..

[22] Khadke S.P., Kuvalekar A.A., Harsulkar A.M., Mantri N. (2019). High energy intake induced overexpression of transcription factors and its regulatory genes involved in acceleration of hepatic lipogenesis: A rat model for type 2 diabetes. Biomedicines.

[23] Uma B., Hemantkumar S.C., Geetika K., Abul K.N. (2013). Antidiabetic effects of *Embelia ribes* extract in high fat diet and low dose streptozotocin-induced type 2 diabetic rats. Front. Life Sci..

[24] Arshag D.M. (2009). Dyslipidemia in type 2 diabetes mellitus. Nat. Clin. Pract. Endocrinol. Metab..

[25] Rivellese A.A., Maffettone A., Iovine C., Di Marino L., Annuzzi G., Mancini M., Riccardi G. (1996). Long-term effects of fish oil on insulin resistance and plasma lipoproteins in NIDDM patients with hypertriglyceridemia. Diabetes Care.

[26] Montori V.M., Farmer A., Wollan P.C., Dinneen S.F. (2000). Fish oil supplementation in type 2 diabetes: A quantitative systematic review. Diabetes Care.

[27] Ghadge A., Harsulkar A., Karandikar M., Pandit V., Kuvalekar A. (2016). Comparative anti-inflammatory and lipid-normalizing effects of metformin and omega-3 fatty acids through modulation of transcription factors in diabetic rats. Genes Nutr..

[28] Bassett C.M., Rodriguez-Leyva D., Pierce G.N. (2009). Experimental and clinical research findings on the cardiovascular benefits of consuming flaxseed. Appl. Physiol. Nutr. Metab..

[29] Vijaimohan K., Jainu M., Sabitha K.E., Subramaniyam S., Anandhan C., Shyamala D.C.S. (2006). Beneficial effects of alpha linolenic acid rich flaxseed oil on growth performance and hepatic cholesterol metabolism in high fat diet fed rats. Life Sci..

[30] Hendrich S. (2010). (n-3) Fatty acids: Clinical trials in people with type 2 diabetes. Adv. Nutr..

[31] Kus V., Flachs P., Kuda O., Bardova K., Janovska P., Svobodova M., Jilkova Z.M., Rossmeisl M., Wang-Sattler R., Yu Z. (2011). Unmasking differential effects of rosiglitazone and pioglitazone in the combination treatment with n-3 fatty acids in mice fed a high-fat diet. PLoS ONE.

[32] Laila A.E., Noha A.R., Salma M. (2015). Effects of omega-3 fatty acids and pioglitazone combination on insulin resistance through fibroblast growth factor 21 in type 2 diabetes mellitus. EJBAS.

[33] Kaithwas G., Majumdar D. (2012). In-vitro antioxidant and in vivo antidiabetic, antihyperlipidemic activity of linseed oil against streptozotocin-induced toxicity in albino rats. Eur. J. Lipid. Sci. Technol..

[34] Mahmud I., Hossain A., Hossain S., Hannan A., Ali L., Hashimoto M. (2004). Effects of Hilsa ilisa fish oil on the atherogenic lipid profile and glycaemic status of streptozotocin-treated type 1 diabetic rats. Clin. Exp. Pharmacol. Physiol..

[35] Horton J.D., Goldstein J.L., Brown M.S. (2002). SREBPs: Activators of the complete program of cholesterol and fatty acid synthesis in the liver. J. Clin. Investig..

[36] Shimano H., Horton J.D., Hammer R.E., Shimomura I., Brown M.S., Goldstein J.L. (1996). Overproduction of cholesterol and fatty acids causes massive liver enlargement in transgenic mice expressing truncated SREBP-1a. J. Clin. Investig..

[37] Shimano H., Horton J.D., Shimomura I., Hammer R.E., Brown M.S., Goldstein J.L. (1997). Isoform 1c of sterol regulatory element binding protein is less active than isoform 1a in livers of transgenic mice and in cultured cells. J. Clin. Investig..

[38] Shimomura I., Bashmakov Y., Horton J.D. (1999). Increased levels of nuclear SREBP-1c associated with fatty livers in two mouse models of diabetes mellitus. J. Biol. Chem..

[39] Higuchi N., Kato M., Shundo Y., Tajiri H., Tanaka M., Yamashita N., Kohjima M., Kotoh K., Nakamuta M., Takayanagi R. (2008). Liver X receptor in cooperation with SREBP-1c is a major lipid synthesis regulator in nonalcoholic fatty liver disease. Hepatol. Res..

[40] Moon Y.A., Liang G., Xie X., Frank-Kamenetsky M., Fitzgerald K., Koteliansky V., Brown M.S., Goldstein J.L., Horton J.D. (2012). The Scap/SREBP pathway is essential for developing diabetic fatty liver and carbohydrate-induced hypertriglyceridemia in animals. Cell Metab..

[41] Mandave P., Khadke S., Karandikar M., Pandit V., Ranjekar P., Kuvalekar A., Mantri N. (2017). Antidiabetic, lipid normalizing, and nephroprotective actions of the strawberry: A potent supplementary fruit. Int. J. Mol. Sci..

[42] Davidson M.H. (2006). Mechanisms for the hypotriglyceridemic effect of marine omega-3 fatty acids. Am. J. Cardiol..

[43] Sharma S., Black S.M. (2009). Carnitine homeostasis, mitochondrial function, and cardiovascular disease. Drug Discov. Today Dis. Mech..

[44] Cai D., Yuan M., Frantz D.F., Melendez P.A., Hansen L., Lee J., Shoelson S.E. (2005). Local and systemic insulin resistance resulting from hepatic activation of IKK-β and NF-κβ. Nat. Med..

[45] Arkan M.C., Hevener A.L., Greten F.R., Maeda S., Li Z.W., Long J.M., Wynshaw-Boris A., Poli G., Olefsky J., Karin M. (2005). IKK-β links inflammation to obesity-induced insulin resistance. Nat. Med..

[46] Popa C., Netea M.G., van Riel P.L., van der Meer J.W., Stalenhoef A.F. (2007). The role of TNF-α in A in chronic inflammatory conditions, intermediary metabolism, and cardiovascular risk. J. Lipid Res..

[47] Jagannathan-Bogdan M., McDonnell M.E., Shin H., Rehman Q., Hasturk H., Apovian C.M., Nikolajczyk B.S. (2011). Elevated proinflammatory cytokine production by a skewed T cell compartment requires monocytes and promotes inflammation in type 2 diabetes. J. Immunol..

[48] Keller H., Wahli W. (1993). Peroxisome proliferator-activated receptors—A link between endocrinology and nutrition?. Trends Endocrinol. Metab..

[49] Schoonjans K., Staels B., Auwerx J. (1996). The peroxisome proliferator activated receptors (PPARS) and their effects on lipid metabolism and adipocyte differentiation. Biochim. Biophys. Acta.

[50] Boord J.B., Fazio S., Linton M.F. (2002). Cytoplasmic fatty acid-binding proteins: Emerging roles in metabolism and atherosclerosis. Curr. Opin. Lipidol..

[51] Veerkamp J.H., van Moerkerk H.T. (1993). Fatty acid-binding protein and its relation to fatty acid oxidation. Mol. Cell. Biochem..

[52] Newberry E.P., Xie Y., Kennedy S., Han X., Buhman K.K., Luo J., Gross R.W., Davidson N.O. (2003). Decreased hepatic triglyceride accumulation and altered fatty acid uptake in mice with deletion of the liver fatty acid-binding protein gene. J. Biol. Chem..

[53] Wolfrum C., Borrmann C.M., Borchers T., Spener F. (2001). Fatty acids and hypolipidemic drugs regulate peroxisome proliferator-activated receptors alpha—and gamma-mediated gene expression via liver fatty acid binding protein: A signaling path to the nucleus. Proc. Natl. Acad. Sci. USA.

[54] Dutta-Roy A.K. (2000). Transport mechanisms for long-chain polyunsaturated fatty acids in the human placenta. Am. J. Clin. Nutr..

