# Supplementary material for: Synergistic Effect of Omega-3 Fatty Acids and Oral-Hypoglycemic Drug on Lipid Normalization through Modulation of Hepatic Gene Expression in High Fat Diet with Low Streptozotocin-Induced Diabetic Rats
Source: Nutrients. 2020 Nov 27;12(12):3652. doi: 10.3390/nu12123652 (PMC7760711; doi:10.3390/nu12123652)
Supplement: Supplementary file 1 [file nutrients-12-03652-s001.zip › Supplementary Figures.docx]

**Supplementary Figures**

**Supplementary Figure 1**

**Assessment of glucose and lipid profile before diabetes development**

Results are represented as Mean±SE (n = 6 for each group and reactions were carried out in triplicates). *p≤0.05, **p≤0.01 and ***p≤0.001, when compared with the HFDC group (Dunnett’s Multiple Comparisons Test). Each animal of flax and fish groups received 0.5mg/kg b.w. flax and fish oil, respectively throughout the experiment. HC: Healthy control, HFDC: High fat diet control

**Supplementary Figure 2**

**LFT and KFT before diabetes development**

Results are represented as Mean±SE (n = 6 for each group and reactions were carried out in triplicates).***p≤0.001, when compared with the HFDC group (Dunnett’s Multiple Comparisons Test). Each animal of flax and fish groups received 0.5mg/kg b.w. flax and fish oil, respectively throughout the experiment. HC: Healthy control, HFDC: High fat diet control.
