# Supplementary figures and images for: Synergistic Effect of Omega-3 Fatty Acids and Oral-Hypoglycemic Drug on Lipid Normalization through Modulation of Hepatic Gene Expression in High Fat Diet with Low Streptozotocin-Induced Diabetic Rats
Source: Nutrients. 2020 Nov 27;12(12):3652. doi: 10.3390/nu12123652 (PMC7760711; doi:10.3390/nu12123652)

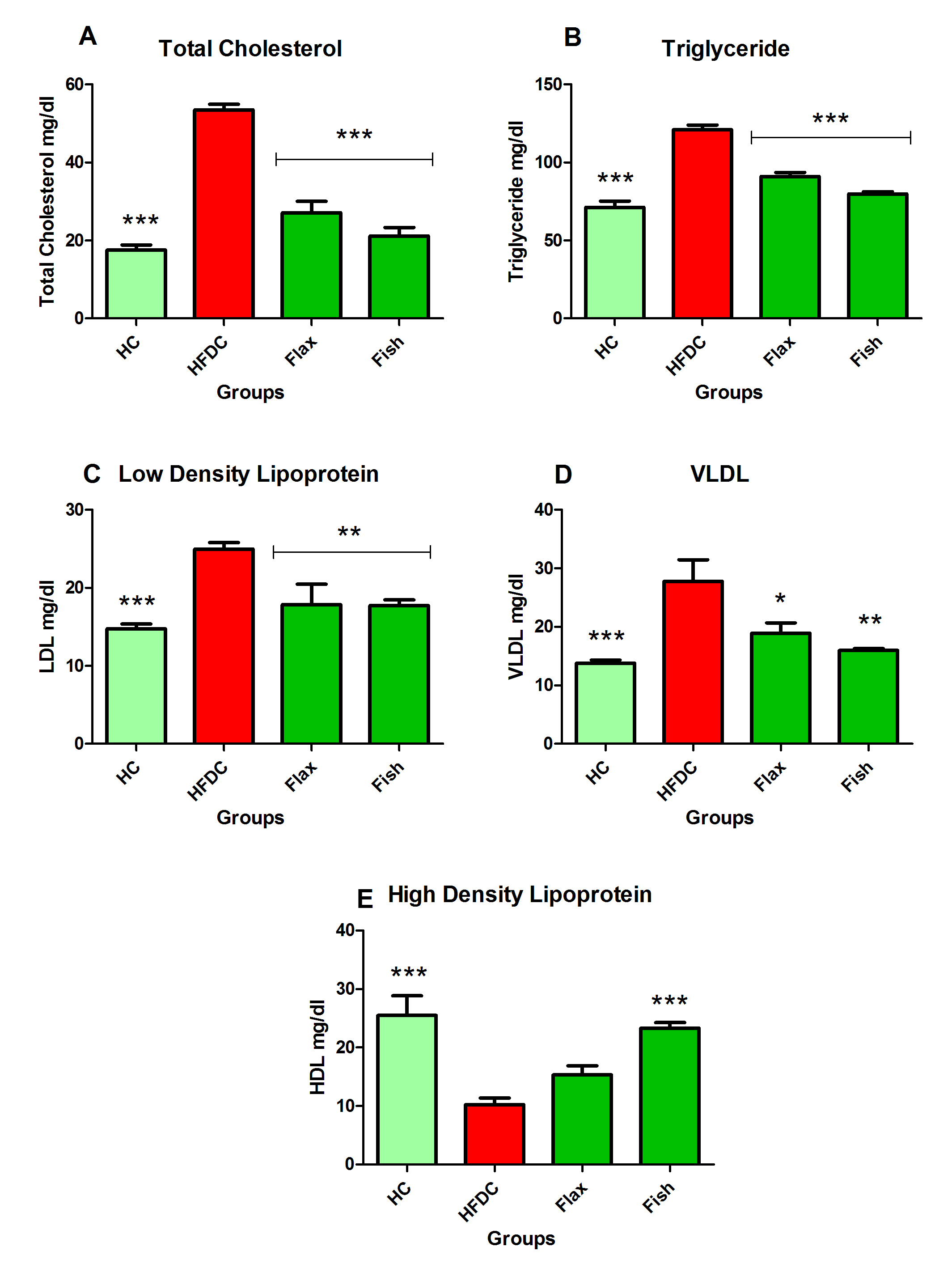

Supplement: Supplementary file 1 [file nutrients-12-03652-s001.zip › Supplementary Figures 1.jpg]

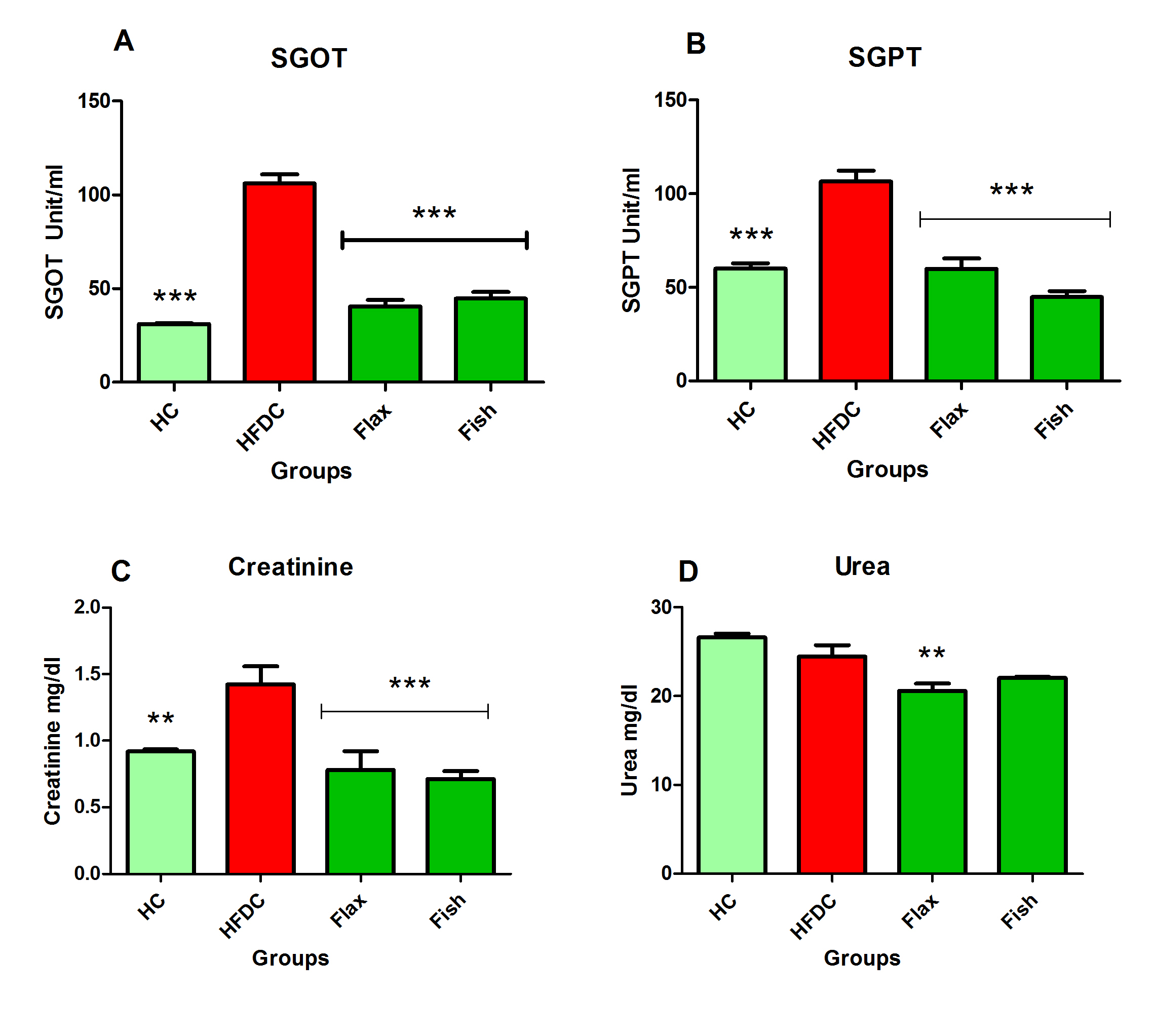

Supplement: Supplementary file 1 [file nutrients-12-03652-s001.zip › Supplementary Figures 2.jpg]
